# Manoeuvring along the edge of breathlessness: an ethnographic case study of two nurses

**DOI:** 10.1186/s12912-016-0148-4

**Published:** 2016-04-27

**Authors:** Maria Omel Jellington, Dorthe Overgaard, Erik Elgaard Sørensen

**Affiliations:** Department of Pulmonary and Infectious Diseases, North Zealand Hospital, Hilleroed, Denmark; Department of Nursing, Metropolitan University College, Copenhagen, Denmark; Department of Clinical Medicine, Aalborg University, Sdr. Skovvej 15, 9000 Aalborg, Denmark; Clinical Nursing Research Unit, Aalborg University Hospital, Sdr. Skovvej 15, 9000 Aalborg, Denmark

**Keywords:** Chronic obstructive pulmonary disease, COPD, Dyspnoea, Dyspnea, Breathlessness, Qualitative research, Ethnographic research, Respiratory nursing, Nurse–patient interaction, Anxiety

## Abstract

**Background:**

There appears to be divergence between nurses’ and patients’ perceptions of dyspnoea onset and on how help should be given. This may affect how nurses understand and assess their patients’ anxiety and the severity of dyspnoea, potentially diminishing their chances of relieving patients’ dyspnoea. The aim of this study was to explore nurse–patient interaction in situations where patients with chronic obstructive pulmonary disease are experiencing acute or worsened dyspnoea in a hospital setting.

**Methods:**

An ethnographic study using participant observation of two nurses’ interactions with six patients, followed by qualitative in-depth interviews with the nurses. Data were analysed in three steps. First, they were coded for identification of preliminary themes. Second, data were regrouped into preliminary themes for focused analysis which led to formulation of themes and subthemes. Third, hermeneutical principles were used as all data were interpreted from the viewpoint of each theme.

**Results:**

Three themes were identified: Manoeuvring along the edge; Dyspnoea within the pattern; and Dyspnoea outside the pattern. They were encompassed by the main finding: Manoeuvring along the edge of breathlessness. The nurses attempted to navigate between implicit and explicit care approaches and to create a sphere for relieving or avoiding further worsening of dyspnoea. Depending on the identified pattern for a particular dyspnoeic episode, nurses attributed different significance to the dyspnoea.

**Conclusions:**

Interacting in dyspnoeic situations places nurses in a dilemma: an implicit approach risk, deriving from exclusion of patients and performing hesitantly; or an explicit negotiation risk, where patients are exhausted and removed from focusing and breathing. The dilemma weakens nurses’ opportunities to relieve or avoid a worsening of the dyspnoea. Likewise, the divergence between nurses’ and patients’ assessment of dyspnoea as within or outside the pattern appears to jeopardize the efficiency of care. Our findings contribute to a deeper understanding of the challenges of respiratory nursing care in general, and the challenges of relieving in-patients’ dyspnoea in particular.

## Background

This study focuses on nurses’ interaction with hospitalized patients suffering from chronic obstructive pulmonary disease (COPD). Although there is an established body of knowledge concerning patients with COPD and their experiences of dyspnoea while in hospital [1–4], little is known about nurse–patient interaction in these situations [5]. We aimed to explore situations in which patients experience acute or worsened dyspnoea. Our findings may contribute to a deeper understanding of the challenges of respiratory nursing care, in particular those faced in relieving inpatients’ dyspnoea.

The world is facing a major health problem as the number of people with COPD increases [6]. Defined as the subjective sensation of difficult breathing [7], dyspnoea becomes the most prominent symptom [8, 9] with the progress of the disease, increasing the risk of acute exacerbation leading to hospitalization [10].

When patients are hospitalized with exacerbation and experiences acute or worsened dyspnoea, they typically depend on nurses’ assessments, planning and actions for relief. As a result, establishing a relationship in the interaction between nurse and patient becomes a key issue in nursing care for these patients [11].

During the last decade, several studies exploring the experience of dyspnoea in relation to exacerbation, from both a patient and a nurse perspective, have appeared [2, 4, 9, 12–15]. They provide evidence of the universality of COPD patients’ experience of fear, panic and anxiety during acute or worsened dyspnoea. Patients see their anxiety as a sign of acute or prolonged respiratory failure, rather than as the cause of dyspnoea - that is, they perceive a dyspnoea–anxiety–dyspnoea cycle [2]. In contrast, nurses consider anxiety to be the cause of the acute disorder, seeing instead an anxiety–dyspnoea–anxiety cycle [14]. Lomborg et al. [13] describe how patients continually have to convince nurses of the severity of their dyspnoeic episodes when receiving assisted personal body care (APBC). They furthermore feel helpless in having to take care of themselves during acute episodes and perceive nurses as reluctant to help them. Lomborg and Kirkevold have established that nurses are only partly aware of their patients’ dyspnoea during APBC. Moreover, they found that in more than 50 % of cases, nurses underestimate the severity of patients’ dyspnoea when compared to patients’ own assessment. Lacking clear and shared interpretations of the patients’ current condition and symptoms, and with no established understanding of the stages of illness, nurses’ performance becomes hesitant [16].

The existing body of knowledge appears to show a divergence between nurses’ and patients’ perceptions of anxiety, dyspnoea and how help should be given - a situation that is potentially detrimental to nurses’ success in relieving their patients’ dyspnoea. It is evident that the way in which nurses interact with their patients during acute or worsened dyspnoea is a complex phenomenon constituted by perceptions created in the interaction. Several elements influence the nurses’ success in interacting with patients to relieve their dyspnoea. Its challenges have already been identified in studies of nurse–patient interaction in APBC contexts [5, 16], but this is the first study to examine nurses’ interaction with patients with COPD hospitalized for exacerbation, the influences of interactions on the dyspnoeic situation, and nurses’ ability to help relieve patients’ dyspnoea.

## Methods

### Aim

The aim was to explore how nurses interact with patients with COPD who experience acute or worsened dyspnoea in a hospital setting. The aim was further illustrated by the research question: “How does the interaction between the nurse and the patient with COPD hospitalized with exacerbation affect the dyspnoeic situation and the ability for the nurse to help relieve patient’s dyspnoea?”

### Design

The study design was inspired by practical ethnographic principles [17–20]. The choice of a focused ethnography method was determined by the distinctness of the studied problem in a small group study [21, 22]. To focus the ethnography, the nurse–patient interaction was defined as a caring relationship that enables the nurse to take appropriate action to help the patient in the situation [23]. Episodes of dyspnoea offer the ideal opportunity for observing the interaction between nurses and patients [16]. To ensure data quality and sensitivity to such episodes a potential dyspnoeic situation was defined as a situation in which the patient; 1) explicitly indicated that they had dyspnoea or laboured breathing; 2) showed signs of increased respiratory rate, prolonged exhalation, or use of auxiliary muscles – indicating that breathing was difficult; or 3) where the nurse took action in an attempt to alleviate a patient’s dyspnoea. Nurses’ actions included the provision of short-acting bronchodilators, repositioning to facilitate breathing, guidance to improve breathing, or emotional support [7]. Or 4) if a nurse indicated that the patient showed anxiety [14] or was unwilling to care for herself, this was also seen as a potential dyspnoeic situation [13, 16, 24].

### Informants and setting

The selection of informants was guided by a wish to obtain rich and nuanced data. We sought to ensure variation in age and professional experience for nurses, while variation in age, severity of disease and experience with hospital admission was essential to the selection of patients [25]. Inclusion criteria for nurses were: more than six months’ experience in the ward; for patients: a Global Initiative for Chronic Obstructive Lung Disease (GOLD) class III or IV diagnosis, i.e., severe or very severe, respectively. The first author’s (MOJ) 6 years of experience from work in a respiratory disease department gave her an intimate knowledge of the studied culture. To avoid the blindness of familiarity [19] and ensure the participant observer’s role as an outsider [18], data were collected in a department at a different university hospital in the Capital Region of Denmark.

A specialized Pulmonary Medicine Department with 600 COPD admissions a year was chosen to insure that the staff had specialized knowledge about caring for patients with COPD. The Danish healthcare system is a public healthcare system predominantly financed through general taxes, and all patients with acute need of hospital treatment for their exacerbation in COPD are admitted. The chosen department consisted of: two outpatient clinics, one hospital outpatient clinic and one inpatient ward. The inpatient ward there the study was carried out has 28 beds spread over 14 rooms. At the ward the nurses practice total patient care for a number of patients and might receive new patients during the day. Furthermore the nurses are responsible for coordinating different tasks involving their own patients, assigned to nursing staff of different levels. Access to the ward was granted by the head nurse. The selection of nurse participants was negotiated between the first author (MOJ) and the head nurse, the latter ensuring that the selected nurses were assigned to patients who met the defined inclusion criteria. Two female nurses and six female patients participated. The nurses were aged 25 and 38 years, with 1 and 7 years of professional experience with COPD patients respectively. Patients were between the ages of 74 and 87, with at least two and less than 12 hospital admissions.

### Data collection

Data were collected through participant observation and interviews by the first author (MOJ).

The nurses’ interactions with patients were observed for 32 h over 4 days. The nurses were followed throughout day shifts to observe a representative range of activities and nurse–patient interactions, such as the observation of patients, medicine administration, communicating about symptoms of breathlessness, assisting with personal body care, receiving patients to initiate treatment of acute non-invasive ventilation (NIV), caring for patients already in NIV treatment, and mobilizing patients. According to Spradley [18] a moderate participation position was chosen trying to maintain a balance between being an insider and outsider, and between participant and observer. On one occasion the observer turned from moderate participation to active participation due to the patients’ condition. Grand tour and mini-tour observations alternated [18]. Grand tours primarily consisted of observation aimed at gaining an overview of the situation. Mini-tour observations involved a more in-depth study of aspects identified on the basis of previous data and findings, such as space, actors, activity, object, act, event, time, goal and feeling [18]. Based on the highly condensed “naive” field notes made during and immediately after observations, complete and expanded notes were prepared after the day’s observations and typewritten verbatim [18]. During the taking of field notes, measures were taken to protect the nurse’s and patient’s formal and informal oral accounts from tampering by interpretation and simplification. To prevent confusion as to the provenance of remarks and observations, each informant was assigned a code. Moreover, to avoid summarization and generalization, descriptions of the observed situations were made as concrete as possible [18]. Furthermore, a reflective journal was kept to ensure awareness of the ethical aspects and to retain ideas for analysis and to safeguard against jumping to conclusions [18].

Following the participant observation period, an in-depth interview [26] was conducted with each of the two nurses. The 1-hour interviews were inspired by a semi-structured guide allowing for both standard questions and questions based on field observations. A recurring question was: “During our days together, did any situation occur in which your patients experienced breathlessness?” A question generated from the observation was: “You asked most of the patients how they experienced their breathing the first time you saw them. What are your thoughts about this?” The interviews were tape-recorded and subsequently typewritten verbatim.

### Data analysis

The data consisted of field notes and interviews. The first author (MOJ) prepared verbatim transcriptions of all notes and interviews. The text was then analysed (MOJ and EES) in accordance with Hammersley and Atkinson [19] in order to identify meaning structures and significant relationships beyond what was observed and said. The text was reviewed around ten times to identify patterns and generate preliminary themes. Coding of data according to Kvale and Brinkmann [26] identified four preliminary themes: *Managing dyspnoea, Collecting information about dyspnoea, Framework, structure and time* and *Types of dyspnoea.* Table 1 illustrates the analytical process and the generation of preliminary themes. After regrouping of data within the preliminary themes, they were analysed to construct four new sets of data, focusing on identifying similarities, differences and interrelations [19, 20]. This led to a further regrouping into three themes: *Manoeuvring along the edge, Dyspnoea within the pattern* and *Dyspnoea outside the pattern*. See Table 2 for an example of the analysis and generation of themes and subthemes from preliminary themes. Third, hermeneutical principles were applied in the interpretation of data from the viewpoint of each theme [26]. This involved several revisions of the raw data with a view to each of the themes. The main theme, *Manoeuvring along the edge of breathlessness,* was finally formulated*.* Table 3 shows the relationship between the main finding, the themes and their subthemes.

**Table 1:** Moving from empirical data to preliminary themes

Step 1:	
Empirical data	Preliminary themes
“The nurse (Inf. 2) tells me (the observer) that she notices that the patient (Pt. 3) had breathlessness, but that she was still able to do a lot of talking. Her breathing stabilised quickly, indicating to the nurse that the patient could handle the breathlessness in that situation. The nurse furthermore explains that the patient paused several times during their conversation, which told her that the patient was in control of her breathing”	Managing dyspnoea
“The nurse (Inf. 1) is explaining her actions in a situation where the patient (Pt. 5) wanted more water even though she had a glass already. “It’s like this –if I tell the patient: “Now, look, you already have a glass of water – that’s enough, you don’t need another one.” That would agitate her, making her breathlessness even worse. So in that situation, I do the same as in any other breathlessness situation – I try to support her in creating order out of chaos, because it allays her concerns and gives tranquillity. Bringing her the extra glass of water helps achieve that.”	
“The pulse oximeter is a very important tool for us because COPD patients, they really can cheat you. The patient will be sitting, eating breakfast and thinking he is doing really well, and then, when you put on the pulse oximeter, the saturation is only 76 %. So you could say that it’s the last part of an overall picture of the patient.” (Inf. 1)	Collecting information about dyspnoea
The nurse (Inf. 2) enters the patient’s room (Pt. 1) with a pulse oximeter. She places the instrument on the patient’s finger, saying: “93.94 % – now, that’s good, I’ll decrease the oxygen level to half a litre.”	
“There was a situation during a night watch where I experienced I didn’t have the time to stay with a COPD patient while she was breathless. I gave the patient a face mask with medicine and then had to leave her. When I came back, the patient’s breathlessness was even worse and beyond her control. Helping her calm down again was really tough. It was unpleasant, both for the patient, but also for me.” (Inf. 2)	Framework, structure and time
“She (Pt. 1) quickly gets exertional dyspnoea. As I see it, she is extremely discouraged by it, to the point where she stays in bed most of the time.” (Inf. 2)	Types of dyspnoea
“Actually, I noticed immediately as I entered the room that she (Pt. 4) was gasping for her breath, and I thought this is a good sign, because it [her breathlessness] must have been from walking to the toilet and back, and I thought to myself, she needs an inhalation, quickly.” (Inf. 1)	
“You sometimes have a patient with a dyspnoea that has completely taken over, so that they cannot find head or tail of anything. They have red flushing cheeks, they are sweating, hot … and their breathlessness has left them completely out of control. They get anxious, become powerless and have no idea what to do. That’s really uncomfortable to watch, I think.” (Inf. 2)

**Table 2:**
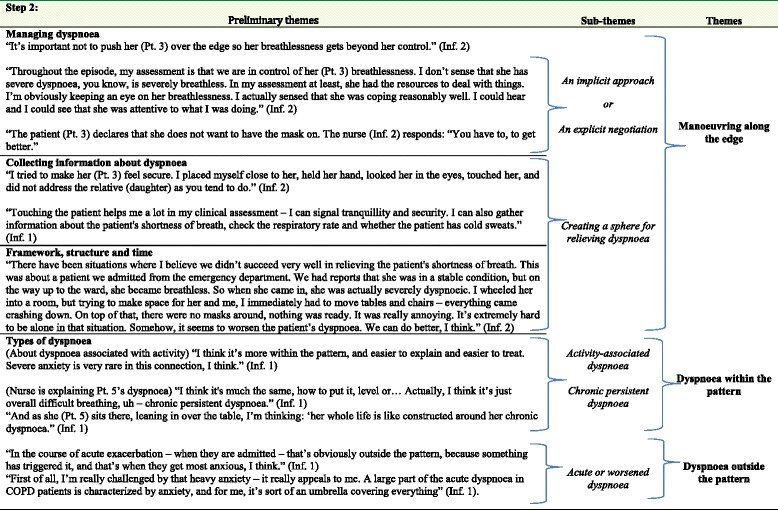
Moving from preliminary themes to subthemes and themes: a process example

**Table 3:** Relationships between main finding, themes and subthemes

Step 3: Identification of main theme by reading empirical data from viewpoint of each theme					
Main finding	Manoeuvring along the edge of breathlessness				
Themes	Manoeuvring along the edge		Dyspnoea within the pattern		Dyspnoea outside the pattern
*Subthemes*	*An implicit approach or explicit negotiation*	*Creating a sphere for relieving dyspnoea*	*Activity-associated dyspnoea*	*Chronic persistent dyspnoea*	*Acute dyspnoea*

## Results

The main finding, *Manoeuvring along the edge of breathlessness,* was based on three themes (Table 3). The first theme, entitled *Manoeuvring along the edge* appeared from observations and interviews where nurses communicated and were observed to be continuously manoeuvring in their interaction with patients. The second and third themes, entitled *Dyspnoea within the pattern* and *Dyspnoea outside the pattern*, respectively, emerged as a reflection of nurses’ attribution of different significance to patients’ dyspnoea, depending on which pattern of dyspnoea they assessed the patient to have, in the specific dyspnoeic situation.

The findings section describes the relationship between the main finding and the three themes. Observations or statements from interviews are given reference numbers for the two female nurses (Inf. 1 and Inf. 2) and for the six female patients (Pt. 1–6).

### Manoeuvring along the edge

When nurses talked about and interacted in situations with patients, they described and were observed to be in a constant state of alertness, trying to avoid provoking or further worsening the patient’s dyspnoea. This was facilitated by manoeuvring along the edge, an approach in which nurses continuously tried to be or act to ensure that patients were cared for without triggering or further worsening their dyspnoea. This approach was reflected in the following response from the nurse after she had assisted a patient with personal body care:

“*She* [Pt. 1] *appeared to be breathless, but she also talked a lot, so I sensed she could handle the breathlessness. She stabilized quickly and managed to control her breath on her own by taking breaks during our conversation. It’s important not to push her over the edge so that the breathlessness moves out of her control*” (Inf. 2).

Manoeuvring along the edge contained two different aspects: *An implicit approach or explicit negotiation* and *Creating a sphere for relieving dyspnoea* (Table 3).

#### An implicit approach or explicit negotiation

When nurses interacted with their patients an implicit approach was verbalized and observed. One nurse (Inf. 1) choose not to discuss her deliberations and actions to relieve or avoid further worsening dyspnoea in observed episodes with Patient 5 and 6. The nurse was asked to elaborate on the described episode and responded: *“In such a situation they are even less able to relate to what I’m thinking. Here it’s important just to help and support, not to explain”* (Inf. 1).

When the nurses assessed patient’s dyspnoea to be acute, the chosen approach was explicit negotiation. Nurses would then negotiate and explain their interventions to their patients. This is illustrated by the following observation in which the nurse initiated acute NIV treatment: “*The patient* [Pt. 3] *declares that she doesn’t want to have the mask on. The nurse* [Inf. 2] *answers, “You have to, to get better”.* [Later, the patient reiterates] *“I want the mask off.” The nurse responds: “You can’t yet – the blood sample I’m taking now will show if the treatment is working”.*

Whether nurses used the implicit approach or chose explicit negotiation in a situation seemed to influence how well she was able to manoeuvre along the edge in the attempt to relieve or avoid further worsening the patient’s dyspnoea. Episodes in which the nurses were successful in relieving patients’ dyspnoea or avoid further worsening were observed and discussed, but also less successful situations where difficulties occurred. The latter kind of situation seemed to occur when the nurse predominantly used the implicit approach and appeared to inhibit nurses from manoeuvring along the edge. This was illustrated by an observation of a nurse giving personal body care to a bedridden patient:

*“The patient* [Pt. 3] *says: ‘I don’t have the energy.’ The nurse* [Inf. 2] *answers: ‘We’ll take it step by step’. After the nurse has only just started to wash the patient’s back, the patient says: ‘No more.’ The nurse then washes the patient’s chest and helps her into a clean shirt. The patient asks for a drink of water. The nurse helps with the water. The patient exhales once more. Rattling sounds are heard, especially during exhalation. How breathless she sounds! The nurse performs intimate body care. She asks the patient what panties she would like to wear.”*

#### Creating a sphere for relieving dyspnoea

Nurses said that in safeguarding the patient’s sense of security, their top priority was to create a sphere for relieving dyspnoea. They saw their manoeuvring to provide security as a multi-faceted effort involving a bodily approach and a specific structural framework. Both aspects were apparent when the nurses recalled episodes in which they felt they had helped relieve the patient’s dyspnoea. By using the bodily approach when manoeuvring in the dyspnoeic situation, the nurses intended to demonstrate to their patient that they were available in every way, with body and mind. One of the nurses illustrated this when she explained why she used physical contact in a dyspnoeic situation observed with Patient 6: *“I place a hand on their chest and say:’ Try to breathe all the way down into the belly’ – because when you are terrified, you don’t sense anything. Then I use my hands to indicate to them: ‘You stop here, and this is me.’ It helps demarcating them”.* (Inf. 1). The other nurse reflected: *“I believe in sitting down, or getting level with them, holding their hand or holding them – it has a good effect”* (Inf. 2).

The nurses saw the establishing of a specific structural framework as a precondition for their manoeuvring to create an amenable sphere for relieving the patients’ dyspnoea. A number of factors were seen as necessary: time for planning and coordinating tasks, time at the patient’s side, establishing an appropriate physical framework for the patient and themselves, easily accessible equipment, and sufficient space around them. This was expressed extensively by the nurses during their interviews as summarized below by the various quotes from Nurse 1:

*“Having the equipment in the room, that’s very important.” “I really wish that we took staying with the patient the entire time more seriously – that we had the resources to do it. It makes a great difference to lie alone, in a room that is cleared, with enough space around you – with a window that can be opened, and you don’t lie squeezed in next to the sink.”* (Inf. 1).

Elaborating on the necessary elements of the structural framework in order to manoeuvre, to be able to relieve dyspnoea, the nurse said:

*“It has to do with coordination, planning, equipment and accessibility before the is admitted to the ward, if possible. If the patient is already here, it’s important to have the opportunity to allocate extra resources so that I can maintain calm around the patient and stay with her. Because it’s important for me to stay in contact with the patient.”* (Inf. 1).

The opportunity and ability to ensure the specific structural framework and create a bodily approach appeared to have consequences for the nurses’ ability to relieve or avoid a worsening of the dyspnoea. This was illustrated by a dialogue between the nurse and a patient who had been stabilized by the NIV treatment:

*“The nurse* [Inf. 2] *asks: ‘Are you at all interested in having the NIV mask on?’ The patient* [Pt. 3] *answers: ‘Good Lord, no!.’ The nurse: “What do you wish?” The answer is unintelligible. The nurse says: ‘We will take it slow. I can hear that you are out of breath.’ The nurse is then interrupted by a colleague and leaves the room.”*

In the interview with Nurse 1, the consequences of a lack of a specific structural framework to ensure a bodily approach was described in further detail:

*“When the physical settings are not in order … the patient is really dyspnoeic, you have to move chairs and tables … there isn’t enough space, and the masks are not there – nothing is ready. That’s horrible. It’s difficult to signal peacefulness when you have to focus on all kinds of practical stuff. There are too many disturbances that inhibit you from helping the patient with his dyspnoea.”* (Inf. 1).

### Dyspnoea within the pattern

The theme entitled dyspnoea within the pattern emerged as a reflection of the nurses’ identification of two different patterns of dyspnoea. They were observed to be present and act in a particular way, depending on which of the two patterns they assessed the patients’ dyspnoea to be in the specific situation.

Reflecting on a dyspnoeic situation involving Patient 4, the nurse said: *“I think* [the patients breathlessness] *kind of falls within the pattern”* (Inf. 1). Dyspnoea perceived to be within the pattern appeared to encompass two types of breathlessness, and were identified by the nurses as: *Activity-associated dyspnoea* and as *Chronic persistent dyspnoea*.

#### Activity-associated dyspnoea

The essential feature of the nurses’ perception of activity-associated dyspnoea was its relative mildness, as illustrated by one of the nurse’s recollection of a dyspnoeic situation with Patient 3*: “I enter the room and see she’s breathless; she has dyspnoea. I’m told that her oxygen saturation is 88 %. I give her a mask and quickly manage to put her at ease”* (Inf. 2).

The other nurse elaborates on the characteristics of activity-associated dyspnoea in the interview: *“It is really very easy to deal with and the course is quite calm.”* (Inf. 1). The nurses perceived activity-associated dyspnoea as predictable, manageable and relatively easy to relieve. This is illustrated by remarks from Nurse 2 when she elaborated on a dyspnoeic situation with Patient 1: *“Again, I placed my hand on her wrist, and said to her: “We can handle this – now I’ll give you a mask, and you’ll feel better about your breathing.” I think five minutes passed with the mask over her mouth – then she was all right”* (Inf. 2).

#### Chronic persistent dyspnoea

While the nurses also perceived chronic persistent dyspnoea as relatively mild, they saw it as rather more difficult to handle in comparison to activity-associated dyspnoea. Describing Patient 5’s dyspnoea during the interview, Nurse 1 commented:

*“The advantage for patients with chronic persistent dyspnoea, in general, is that they don’t have big fluctuations of their dyspnoea. They don’t experience dyspnoea the way others* [patients] *do. Generally, these patients can sometimes be difficult to help, because their breathing is persistently bad. This can be difficult to deal with, and I have a tendency to just accept it”* (Inf. 1).

Applicable to both activity-associated dyspnoea and chronic persistent dyspnoea was that, the nurses perceived the patients’ anxiety as manageable, reasonably simple to relieve, and as something most patients were very good at coping with. In her interview the nurse explained: *“As long as the dyspnoea is within the pattern, the anxiety won’t last long – it’s not so pronounced. Most COPD patients are extremely good at coping with anxiety, when their dyspnoea is within the pattern”* (Inf. 1). If the anxiety turned out to be more severe than anticipated, the nurses seemed to interpret it as an existential anxiety rather than one related to the dyspnoea. *“Of course, they will worry if they can’t do something today they could do yesterday. That does not necessarily relate to their dyspnoea. It could simply be a fear of life”* (Inf. 1).

### Dyspnoea outside the pattern

Dyspnoea outside the pattern was the other pattern for dyspnoea that nurses identified and directed their actions against as defined by the nurse during the interview: *“When they come in with acute exacerbation, it* [the dyspnoea] *is clearly outside the pattern”* (Inf. 1). Dyspnoea outside the pattern contained one type of breathlessness: *Acute dyspnoea*.

#### Acute dyspnoea

According to the nurses, the essential features of acute dyspnoea are sudden onset or admission for acute exacerbation of COPD. As explained by the nurse during the interview: *“If they suddenly experience breathlessness in a situation where they’re just sitting quietly or something just happens.”* (Inf. 1). In these situations, the nurses would anticipate severe anxiety. They responded to what they perceived as an appeal for care and appeared to become very attentive to their patients, as reflected in remarks by both nurses during their interviews: *“They will wake up, become anxious – their respiration is fast and shallow, the skin clammy, and they’ll look at you as if this is the end, now they are dying”* (Inf. 2). *“First of all, I’m really challenged by that heavy anxiety – it really appeals to me. A large part of the acute dyspnoea in COPD patients is characterized by anxiety, and for me, it’s sort of an umbrella covering everything”* (Inf. 1).

A divergence of views which seemed to be linked to patients’ and nurses’ different perceptions of the severity of their dyspnoea was observed. This is illustrated by the nurse’s unsolicited interpretation of remarks from Patient 5 in a dyspnoeic situation:

*“She told* [the doctor and me] *how her saturation had been at 87 % and she had felt so poorly yesterday morning and had needed a lot of masks. I corrected her, saying it was 88 %, and that she had recovered quickly after two masks. She then said she wanted a pulse oximeter at home so she could prove how sick she is. Those were her words. I just thought, “Come on”. On the one hand I feel sorry for her, and on the other hand, I lose my motivation to dialogue with her. Oh, my goodness, I hate to say this, but this is how I feel.”* (Inf. 1).

## Discussion

The aim of this study was to explore nurse–patient interaction in situations where patients with COPD experience acute or worsened dyspnoea in a hospital setting. The findings were grouped into three themes: *Manoeuvring along the edge, Dyspnoea within the pattern,* and *Dyspnoea outside the pattern.*

In the study we found the nurses to be in a constant position of manoeuvring in their attempt to avoid further worsening of the patients’ dyspnoea. This finding extends Lomborg and Kirkevold’s [5] work on nurses giving assisted personal body care (APBC) to in-patients with COPD. Those authors define “curtailing” as the challenging and delicate balance nurses achieve by protecting patients from dyspnoea while promoting their capabilities. Our findings suggest that the nurses give priority to protecting patients from dyspnoea, not only during APBC, but in all interactions in which they sense a risk of worsened dyspnoea. The Nurses’ typical approach of manoeuvring along the edge seems fraught with difficulties. In line with Lomborg and Kirkevold’s [16] finding that nurses underestimate the severity of patients’ dyspnoea in more than half of the cases, we witnessed situations in which the nurses’ failed to make a correct assessment, which caused them to perform hesitantly.

The nurses consider manoeuvring along the edge as primarily an implicit approach, based on their perception that patients are unable to relate to the nurses’ assessment while dyspnoeic. They furthermore indicate that the implicitness of the approach is essential for their interaction with patients in dyspnoeic situations. To prevent further worsening the dyspnoea, the nurses avoid sharing their reflection with the patients and avoid seeking the patients’ own assessment of dyspnoea, a view that corresponds well with findings that patients feel that to survive an attack, they are compelled to focus completely on their breathing [12]. By taking an implicit approach, the nurses are deprived of the opportunity to include the patients in their assessment of episodes, which has been shown to lead to a hesitant performance [16]. The choice to manoeuvre along the edge thus places the nurses in an obvious dilemma between either involving or protecting the patients. This dilemma is compounded by what Kvangarsnes and co-workers [24] calls patients’ altered perception of reality. During acute exacerbation, they found, the patients’ ability to understand the seriousness of their situation is constrained. While our study provides no answers to how this problem should be handled, it appears to weaken the opportunities for the nurses to relieve or avoid a worsening of the dyspnoea experienced by their dyspnoeic patients with exacerbated COPD. Observational studies for further exploration of this dilemma are recommended.

We found that when the nurses assess the dyspnoea to be within the pattern, they perceive the patients’ anxiety as short-lived and mild. When their assessment points to dyspnoea outside the pattern, they characterize it as rampant, perseverant and as a natural consequence to the acute dyspnoea.

Our identification of different types of anxiety corresponds with earlier research into patients’ view of anxiety. In a Swedish study, it was found that GOLD class III and IV patients experience acute respiratory distress as a clear instance of anxiety, bordering on panic [27]. In research on COPD patients’ experience of sleep, two types of anxiety were found: 1) everyday anxiety and fear of breathlessness dominating the person’s daily experience, limiting physical and social activities and impairing the quality of life, and 2) panic or additional anxiety provoked by a sudden onset of breathlessness [28]. Bailey identified different perceptions of anxiety as communicated by patients through their stories about near-death and shadow-of-death [1]. Across studies, it appears that patients’ and nurses’ have consistent perceptions of the types of anxiety, depending on the severity of breathlessness. We believe that conformity between patients’ and nurses’ assessments of the patients’ dyspnoea being within or outside the pattern is essential to nurse–patient interaction. Where divergencies between their assessments prevail, the consequence seems to be that nurses’ opportunity to relieve or avoid further worsening of the dyspnoeic situation is diminished. It also appears that patients’ anxiety reflects such divergencies.

Bailey [2] defines an anxiety–dyspnoea–anxiety circle as the starting point for nurses’ interpretation of patients’ dyspnoea. The same author and her co-workers [14] found that nurses’ care strategies were based on a perceptual template according to which anxiety is always the cause of the patients’ dyspnoea, and thus of their admission to hospital. This study has added to our understanding of these aspects in that our findings provide no evidence that the nurses always see anxiety as the cause of dyspnoea. When the nurses’ expectations correspond to the perceived level of anxiety, they do not attribute dyspnoea to anxiety. This is true, whether or not the nurses assess the dyspnoea to be within or outside the pattern. However, when the nurses meet with unexpectedly severe anxiety, it appears that they understand anxiety as a precursor to dyspnoea. This was the case when we observed the nurses to assess dyspnoea to be outside the pattern. In such situations, they seem to perceive dyspnoea as disproportionate to the situation and a hindrance to patients’ opportunity to function. We therefore argue that in their clinical interpretation of dyspnoeic situations, nurses can use the perceived level of anxiety as a marker of whether the patient’s dyspnoea is within or outside the pattern. When nurses experience patients’ anxiety to be disproportionate to the situation, it may indicate a divergence between nurses’ and patients’ interpretation of the pattern in the dyspnoeic situation. In addition to providing a more nuanced understanding of COPD patients’ anxiety in dyspnoeic situations, our findings offer new perspectives on nurse–patient interaction in hospital settings.

We found that a specific-structured framework that enables the nurses to use their preferred bodily approach was prerequisite to their ability to secure a sphere amenable to relieving or avoid a further worsening of dyspnoea. Creating that framework could be difficult for the nurses because of the overall organisation in the ward. Especially due to the number of different staff involved in the patients care and the major focus on efficiency in clinical practice, which means that every bed is occupied all the time. Furthermore interruptions from colleagues seemed to hinder the nurses in creating that sphere. We saw that an interruption made Nurse 1 lose focus in her conversation with a patient in NIV treatment and that she failed to follow up with the patient. Sorensen and Brahe [29] found that nurses’ acceptance of interruptions by colleagues placed them in a dilemma between being accessible and maintaining focus in their work [29]. Our study provides no detailed analysis of the impact of interruptions on the nurses’ ability to maintain a bodily approach and relieve dyspnoea in the interaction with the patients. Further research is thus needed to understand how interruptions affect the nurse–patient interaction during dyspnoea episodes, and to identify effective interventions to prevent interruptions.

### Rigour

The credibility of this study is enhanced by the use of a variety of data sources, such as participant observation, formal and informal oral accounts, field notes, and qualitative interviews [25]. The chosen position ‘moderate participation’ may have had an impact on the nurses, especially the less experienced nurse. To address this aspect the observer and the nurse reflected on this together afterwards.

To strengthen its trustworthiness, the investigator triangulated by comparing and contrasting divergent views.

To ensure the conformance of the study, a reflexive journal was kept and principled methods of field note taking were applied [18], further supported by audio recordings. In the interviews, the interviewer rephrased or repeated the informants’ statements almost verbatim [26].

In considering the transferability of this study, it should be taken into account that, despite the relatively small sample size, the findings show a high degree of correspondence with existing findings. As a reflexive note from the first author our finding, regarding the nurses’ dilemma between involving or protecting the patient during episodes of dyspnoea, is highly recognizable in her own clinical practice.

Dependability was strengthened by presenting and discussing our findings in a meeting with many of the ward’s nurses. The group agreed that the behavioural patterns and approaches described conformed very well to their experiences in relation to dyspnoea patients. The sample of quotes from the informants’ statements in the findings section strengthens the trustworthiness of the findings. As the first author (MOJ) is a member of the respiratory nursing culture, a certain bias towards the adopted perspectives and interpretations cannot be ruled out [19]. However, the understanding it brought to the study of a new environment enabled identification of outstanding practices and sensitized her to implicit routines, practices, and truisms [30]. On balance, the advantages of choosing a familiar culture in a different setting are believed to exceed the disadvantages.

The limited number of informants was compensated for by the richness of information presented. However, longer field stays and the inclusion of more informants for example from other hospital wards could have helped capture richer and more varied data. For example studies have shown a gender variation in experience of respiratory symptoms [31].

## Conclusions

In manoeuvring along the edge of breathlessness to relieve or avoid further worsening of the patient’s dyspnoea, nurses are facing a dilemma – a dilemma between involving patients and protecting their health. In dyspnoeic situations, nurses are advised to consider the risk that a hesitant performance may exclude the patient from the situation, and that including the patient poses a risk to exhaust the patient and remove focus from breathing. If they fail in this respect, their opportunity to relieve or avoid further worsening of the dyspnoea is ultimately diminished. Our study has shown that when nurses assess dyspnoea to be within the pattern and meet unexpectedly severe anxiety, they tend to see anxiety as a precursor to dyspnoea. The anxiety then becomes out of proportion to the situation.

The understanding of anxiety offered by this study has important implications for clinical practice. In this new light, the expression of anxiety should be used by hospital nurses as a marker of the severity of dyspnoea.

When nurses experience patients’ anxiety to be disproportionate to their clinical condition, they should be sensitive to the possibility that their interpretation of the dyspnoeic situation is out of alignment with their patient’s interpretation. The understanding of anxiety presented here supports nurses in further developing their ability to recognize the subtle signs of divergences between themselves and their patient in the dyspnoeic situation. By being more open about their actions, they will strengthen their ability to reach a common understanding with the patient, which may eventually improve the care provided for their dyspnoeic patients. It may furthermore enable nurses to question and reconsider their care strategies for these patients, ultimately, challenging them to further develop their conceptual templates of dyspnoeic care.

### Ethics and consent to participate

The Capital Region Research Council approved the study protocol (Data Protection Agency, Journal no. 2010-41-4460), as did the Committees on Health Research Ethics for the Capital Region of Denmark (Protocol No. H-1-2013-FSP-50). The study adhered to ethical guidelines for nursing research in the Nordic countries [32]. Having received oral and written information, the participating nurses gave written consent. Informed consent was given by the participating patients at the first meeting and after oral information was provided. In the data collection, the patients’ condition was considered; if they felt too weak to participate, either due to the patients acutely conditions or if they needed strength to deal with discharge.

### Consent to publish

Not applicable.

### Availability of data and materials

Detailed data will not be shared due to the confidential nature of the way that the data has been managed.

## Abbreviations

APBC: assisted personal body care
COPD: chronic obstructive pulmonary disease
GOLD: Global Initiative for Chronic Obstructive Lung Disease
GOLD Class III severe COPD: according to Global Initiative for Chronic Obstructive Lung Disease
GOLD Class IV very severe COPD: according to Global Initiative for Chronic Obstructive Lung Disease
NIV: non-invasive ventilation
